# Are they there yet? Linkage of patients with tuberculosis to services for tobacco cessation and alcohol abuse – a mixed methods study from Karnataka, India

**DOI:** 10.1186/s12913-019-3913-8

**Published:** 2019-02-01

**Authors:** Nagendra Navya, Kathiresan Jeyashree, Akshaya Kibballi Madhukeshwar, Tanu Anand, Abhay Subashrao Nirgude, Badarudeen Mohammad Nayarmoole, Petros Isaakidis

**Affiliations:** 1 Department of Community Medicine, Yenepoya Medical College, Mangaluru, India; 2 Department of Community Medicine, Velammal Medical College Hospital and Research Institute, Madurai, India; 3 Department of Community Medicine, North Delhi Municipal Corporation Medical College Hindu Rao Hospital, New Delhi, India; 4 District Tuberculosis Office, Mangaluru, Dakshina Kannada, Karnataka India; 5 Médecins Sans Frontières, Operational Research Unit, Luxembourg City, Luxembourg

**Keywords:** Tuberculosis, Tobacco use, Alcoholism, Integrated delivery system, Operational research, SORT IT

## Abstract

**Background:**

Tobacco use and alcohol abuse are associated with higher risk of tuberculosis (TB) infection, progression to active TB and adverse treatment outcomes among patients with TB. Revised National Tuberculosis Control Programme (RNTCP) treatment guidelines (2016) require the documentation of tobacco and alcohol use among patients with TB and their linkage to tobacco and alcohol abuse treatment services. This study aimed to assess the extent of documentation of tobacco and alcohol usage data in the TB treatment card and to explore in-depth, the operational issues involved in linkage.

**Methods:**

A convergent parallel mixed methods study was conducted. All new TB treatment cards of adult patients registered under RNTCP between January and June 2017 in Dakshina Kannada district were reviewed to assess documentation. Document review was done to understand the process of linkage (directing patients to tobacco and alcohol abuse treatment services). In-depth interview of health care providers (*n* = 7) and patients with TB (*n* = 5) explored into their perspectives on linkage.

**Results:**

Among 413 treatment cards reviewed, tobacco use was documented in 322 (78%), of whom 86 (21%) were documented as current tobacco users. Sixteen (19%) out of these 86 patients were linked to tobacco cessation services. Alcohol usage status was documented in 319 (77%) cards of whom 71(17%) were documented as alcohol users. Eleven (16%) out of these 71 patients were linked to alcohol abuse treatment services. The questions in the treatment card lacked clarity. Guidelines on eliciting history of substance abuse and criteria for linkage were not detailed. Perceived enablers for linkage included family support, will power of the patients and fear of complications. Challenges included patient’s lack of motivation, financial and time constraints, inadequate guidelines and lack of co-ordination mechanisms between TB programme and tobacco/alcohol abuse treatment services.

**Conclusion:**

Documentation was good but not universally done. Clear operational guidelines on linkage and treatment guidelines for health care providers to appropriately manage the patients with comorbidities are lacking. Lack of coordination between the TB treatment programme and tobacco cessation as well as alcohol treatment services was considered a major challenge in effective implementation of the linkage services.

**Electronic supplementary material:**

The online version of this article (10.1186/s12913-019-3913-8) contains supplementary material, which is available to authorized users.

## Background

Exposure to tobacco smoke increases the risk of tuberculosis (TB) infection, the risk of progression to active TB as well as the risk of relapse and death among TB patients. Moreover, it affects the clinical manifestations, microbiological conversion (sputum smear or culture) and adversely affects the treatment outcomes [1–4]. Lastly, it has been shown that it increases the risk of anti- tubercular drug resistance [5]. Similarly, alcohol abuse increases the risk of acquiring TB infection, causing adverse drug reactions among TB patients undergoing treatment and is associated with adverse treatment outcomes including loss to follow-up, treatment failure and death [6–9].

One fourth of the global burden of TB is from India. Globally, in 2016, an estimated 10.4 million cases and 1.4 million deaths occurred due to TB of which 2.79 million cases and 435,000 deaths had occurred in India [10, 11]. According to Global Adult Tobacco Survey 2 (GATS) in India (2016–17), 28.6% of adults (15 years and above) use tobacco in any form [12]. In 2012, 5.9% of all global deaths or approximately 3.3 million deaths were attributable to consumption of alcohol [13]. In India, the prevalence of heavy episodic alcohol drinking was observed in about 12.9 and 0.7% of men and women above the age of 15 years respectively [13].

There is a formal coordination mechanism between the Technical Working Groups for National TB Programme and Tobacco Control in India [14]. The recent Revised National Tuberculosis Control Programme (RNTCP) treatment guidelines for TB in 2016 included the requirement to document the tobacco use status of TB patients and to integrate brief advice for tobacco cessation in the routine TB care. It also suggested linkage to tobacco cessation services, the Tobacco Cessation Clinics (TCC) or alcohol abuse treatment services and assessment of the tobacco use status at the end of TB treatment [15]. Data on tobacco and alcohol use were added in the new TB treatment card, for the first time since January 2017 in Karnataka and hence it is essential to understand the adequacy of documentation, the process of this linkage and the operational issues in its implementation. Thus, this study aimed to 1) report the extent of documentation of tobacco and alcohol usage data in the TB treatment card 2) explore the process, facilitators and challenges in the linkage of services for tobacco cessation and alcohol abuse from the perspective of health care providers and adult patients with TB under programmatic conditions in Dakshina Kannada district, India.

## Methods

### Study design

This was a convergent parallel mixed methods study including a quantitative (cohort study) to assess the extent of documentation of linkage to tobacco and alcohol treatment services among patients with TB and a simultaneously conducted qualitative (descriptive study) component to understand the enablers and challenges faced by health care providers and patients with TB in doing so [16].

### Setting

#### General setting

The study was conducted in Dakshina Kannada district, one of the 30 districts in the state of Karnataka, India. Tuberculosis units (TUs) are nodal points for TB control activities in the subdistrict. RNTCP services in a district are delivered through the Designated microscopy centres (DMCs) and peripheral health institutions (PHIs) [15]. The district has one Government treatment centre for tobacco and alcohol abuse.

#### Study site

Data was collected from all five TUs (as of June 2017) in the study district. Dakshina Kannada District is the field practice area of the institution where the PI is currently employed and has good working relations with the TB program managers. This would help the results to be of immediate and direct relevance in policy implementation.

### TB, tobacco and alcohol use: Documentation and linkage

When a patient gets registered under the RNTCP, his/ her tobacco use status is enquired and the information is recorded in the new TB treatment card. If the patient is a smoker or tobacco user, he/ she is offered a ‘brief advice’ to quit tobacco use based on the 5As and 5Rs model [15, 17]. The status of tobacco use is assessed at every follow up visit and at the end of treatment, and recorded in the treatment card. On assessment, if the patient has not quit tobacco then s/he is referred to the nearest Tobacco Cessation Clinic (TCC) or quit line or m-cessation initiative. Information recorded in TB treatment card, is sent through existing Health Management Information System (HMIS) under RNTCP [15].

Similarly, history of alcohol use is documented (in the new TB treatment card) when the patient gets registered under RNTCP and he/she is linked (directed) to alcohol abuse treatment services (referred to as “deaddiction” in the new TB treatment card) whenever indicated.

### Study population

#### Quantitative

The study population included all adult patients with TB registered under RNTCP between 1st January 2017 and 30th June 2017. A total of 413 new TB treatment cards of patients registered between 1st January 2017 to 30th June 2017 were available in all the TUs and all these cards were reviewed for the study.

#### Qualitative

Medical Officers (MOs) involved in the treatment of patients registered under RNTCP, health care providers (counselor, psychiatrist) involved in providing tobacco cessation and alcohol abuse treatment services, and patients with TB who were registered for treatment between January 1st and June 30th 2017 were selected by using purposive sampling.

### Data variables, sources of data and data collection

#### Quantitative data collection

TB treatment cards of all registered patients in the five tuberculosis units of Dakshina Kannada district were reviewed by the principal investigator (NN). Data of all registered patients with TB (sociodemographic, TB related, history of tobacco and alcohol use, cessation services, usage status at the end of treatment) was extracted from the TB treatment cards into a structured data collection form**.** However, only cards of patients with drug sensitive TB were reviewed as substance use details of patients with drug resistant TB are not entered in the new TB card. Data validation was carried out by contacting a subset of patients with TB after taking informed consent.

#### Qualitative data collection

Programmatic factors were explored through document review of the TB treatment cards and the TB treatment guidelines 2016 [15] and one to one interviews with key informants involved in providing TB treatment and tobacco cessation and alcohol treatment services. Registered patients with TB were also interviewed to elicit their experiences and opinions about these services. Seven key informants from TB (Medical officers) and tobacco cessation and alcohol abuse treatment services (Psychiatrist and Counselor) willing to participate were purposively selected and interviewed. The interviews had a mean (range) duration of 12.2(6–32) minutes. The interviews explored the processes, perceived facilitators and challenges faced by them in linkage to tobacco cessation and alcohol treatment services as well as their suggestions to improve it. Five patients, who were willing to participate were purposively selected and interviewed to explore their experiences in accessing care. After 7 interviews with health care providers and 5 interviews with patients with TB no new information was being obtained hence data collection was terminated.

The Principal Investigator (NN) conducted the interviews. She is a faculty member (MBBS, MD in Community Medicine) in a medical college located in the study area, speaks the local languages (Kannada, Tulu), not involved in implementation of RNTCP and is trained in qualitative research methods.

Key informant Interviews (KII) were done at the place, date and time most convenient to the participants. The Principal Investigator (PI) conducted one-to-one interviews using an interview guide (Additional file 1) with open ended questions after obtaining their consent to participate in the study and audio-recorded the interviews. The interview was conducted in a place were only the interviewer and interviewee were present during the interview, and the interviewees were assured that their identification would not be revealed to anybody, thus ensuring anonymity, privacy and confidentiality.

The Principal Investigator conducted telephonic interviews with consenting patients in local language at a time convenient to them. The consent was sought in two stages. The community health worker asked the purposively selected patients whether they consent to be interviewed over the phone and obtained a written informed consent from them. At the second stage, PI called the patients and after obtaining a verbal confirmatory consent for audio recording of the interview, proceeded to the interview using an interview guide with open ended questions. At the end of the interview the participants were debriefed and given opportunity to clarify the same.

The findings of the in-depth interviews were discussed with the co-investigators and questions were modified accordingly for the subsequent interviews. Transcripts prepared were reviewed by the co investigators.

### Analysis and statistics

#### Quantitative

Quantitative data collected was double- entered and, validated using EpiData entry v.3.1 and analyzed using EpiData analysis v. 2.2.2.178 EpiData Association, Odense, Denmark. Key analytic outputs included the number and proportion of patients with TB whose tobacco and/ or alcohol usage status is documented at registration and end of treatment, the number and proportion of TB patients documented as tobacco and alcohol users, the number and proportion of tobacco and alcohol abusers referred to cessation/ treatment services. Association of linkage to tobacco and/ or alcohol abuse treatment services with various socio- demographic and clinical factors was assessed using Relative Risks (RR).

#### Qualitative

Audio recorded interviews were transcribed in English on the same day of the interviews. Manual thematic analysis was used to analyze the data. The initial coding and theme generation was done by the Principal investigator (NN) and reviewed by a second investigator (JK and PI). Any difference between the two was resolved by discussion. Similar basic themes were grouped as organizing themes and then into a global theme, utilizing a thematic network analysis method as described by Attride-Stirling [18]. The findings are reported by using ‘Consolidated Criteria for Reporting Qualitative Research [19].

## Results

### Quantitative

Of the total 413 patients included in the study 278 (67.3%) were males, 234 (56.7%) were residing in rural areas and 254 (61.5%) belonged to below the poverty line (BPL- income limit less than Indian Rs 27,000 per annum) income group. The mean age of the study participants was 42.6 years; Pulmonary TB accounted for 335 (81.1%) of the cases and 320 (77.5%) were new TB cases (Table 1).

**Table 1:** Clinical and demographic profile of adult patients with TB^a^ (*N* = 413) between January and June 2017

Variable	Frequency/Mean	Percentage/SD
Age in years	42.6	14.7
Gender		
Male	278	67.3
Female	130	31.5
Not documented	5	1.2
Area		
Rural	234	56.7
Urban	109	26.4
Not documented	70	16.9
Socio economic status		
Above poverty line	84	20.3
Below poverty line	254	61.5
Not documented	75	18.2
Site of disease		
Pulmonary	335	81.1
Extra pulmonary	78	18.9
Type of Patient		
New	320	77.5
Recurrent	41	9.9
Transferred in	3	0.7
Treatment after loss to follow up	20	4.8
Treatment after failure	10	2.4
Others	16	3.9
Not documented	3	0.7
Regimen		
New	271	65.6
Previously treated	69	16.7
Not documented	73	17.7
HIV status		
Reactive	20	4.8
Non reactive	383	92.7
Not documented	10	2.4
Treatment Outcome		
Cured	168	40.7
Completed treatment	110	26.6
Treatment failed	13	3.1
Lost to follow up	24	5.8
Died	28	6.8
Not evaluated	3	0.7
Not documented	67	16.2

^a^*TB* Tuberculosis, *RNTCP* Revised National Tuberculosis Control Programme, *SD* Standard Deviation

Tobacco use was documented in 322 (78%) of the TB treatment cards reviewed. Among the 86 (21%) patients documented as current tobacco users, 16 (19%) were linked to tobacco cessation services while for 46 (53.5%) of them linkage was not documented in their treatment cards.

Alcohol use status was documented for 319 (77%) of the TB patients. Among the 71(17%) documented as alcohol abusers, 11 (16%) were linked to alcohol treatment services.

Of the 413 TB patients, 47 (11.4%) were documented as users of both tobacco and alcohol, out of which 7(14.9%) were linked to tobacco cessation and 9 (19%) to alcohol abuse treatment services.

**Fig. 1:**
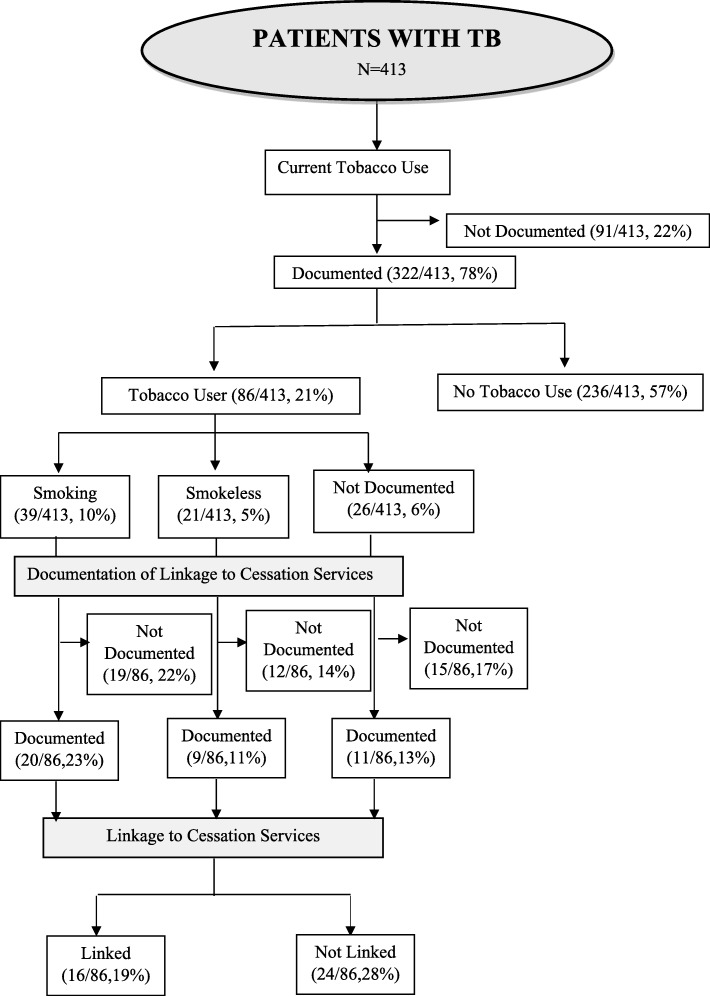
Documentation of Tobacco Use and linkage to cessation services among adult patients with TB. The extent of documentation of current tobacco use status and linkage to cessation services among adult patients with TB was assessed by document review of the new TB treatment cards which is depicted in the flowchart. Data showed that 78% of the cards had documentation of tobacco use status and 19% of the users were linked to cessation services

**Fig. 2:**
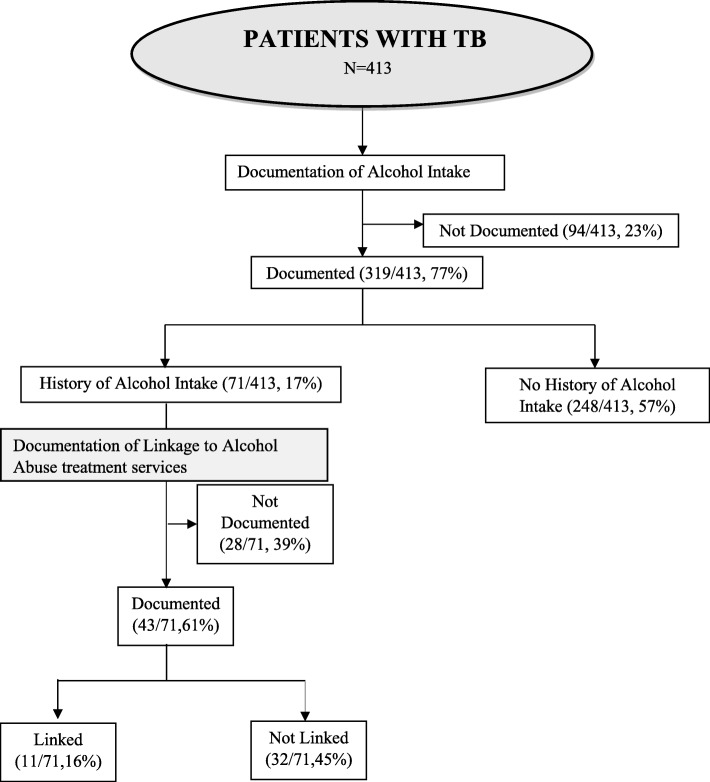
Documentation of alcohol intake and linkage to alcohol abuse treatment services among adult patients with TB. The extent of documentation of alcohol intake and linkage to alcohol abuse treatment services among adult patients with TB was assessed by document review of the new TB treatment cards which is depicted in the flowchart. Data showed that 77% of the cards had documentation of history of alcohol intake and 16% of the patients with history of alcohol intake were linked to alcohol abuse treatment services

The cascade of patients from registration under RNTCP to the linkage to tobacco and alcohol abuse treatment services (as assessed at the end of TB treatment) are shown in Figs. 1 and 2 respectively.

Factors associated with linkage to tobacco cessation services and alcohol abuse treatment services are presented in (Tables 2 and 3). Patients with pulmonary TB were more likely to be linked to alcohol abuse treatment services than extra pulmonary TB and this difference was found to be statistically significant (*p* = 0.01) (Table 3). Patients linked to alcohol abuse treatment services were more likely to be also linked to tobacco cessation services and this difference was found to be statistically significant (*p* = 0.02). (Table 2).

**Table 2:** Association of various factors with linkage to tobacco cessation services among adult patients with TB

Variables	Linkage to tobacco cessation Services n (%)		
*N* = 86	Yes	No	
Gender (*N* = 39)			
Male	14 (38.9)	22 (61.1)	*P* = 0.84
Female	1 (33.3)	2 (66.7)	
Area (*N* = 36)			
Rural	11 (35.5)	20 (64.5)	*P* = 0.84
Urban	2 (40)	3 (60)	
Socio economic status(*N* = 32)			
Above poverty line	2 (33.3)	4 (66.7)	*P* = 0.8
Below poverty line	10 (38.5)	16 (61.5)	
Site of disease (*N* = 40)			
Pulmonary	14 (37.8)	23 (62.2)	*P* = 0.32
Extra pulmonary	2 (66.7)	1 (33.3)	
Regimen type (*N* = 37)			
New	11 (42.3)	15 (57.7)	*P* = 0.73
Previously treated	4 (36.4)	7 (63.6)	
Type of Tobacco use (*N* = 29)			
Smoking	9 (45)	11 (55)	*P* = 0.07
Smokeless	1 (11.1)	8 (88.9)	
Tobacco use at end of treatment (*N* = 8)			
Quit	3 (50)	3 (50)	*P* = 0.2
Not Quit	0	2 (100)	
History of Alcohol Intake(*N* = 40)			
Yes	7 (35)	13 (65)	*P* = 0.57
No	7 (41.2)	10 (58.8)	
Not documented	2 (66.7)	1 (33.3)	
If History of Alcohol intake present, linked to alcohol abuse treatment services (*N* = 14)			
Yes	3 (75)	1 (25)	*P* = 0.02
No	1 (10)	9 (90)	

*TB* Tuberculosis, *RNTCP* Revised National Tuberculosis Control Programme

**Table 3:** Association of various factors with Linkage To Alcohol Treatment Services Among Adult Patients with TB

Variables	Linkage to Alcohol Abuse Treatment Services n (%)		
*N* = 71	Yes	No	
Gender(*N* = 41)			
Male	10 (25.6)	29 (74.4)	*P* = 0.4
Female	0	2 (100)	
Area (*N* = 37)			
Rural	8 (25)	24 (75)	*P* = 0.2
Urban	0	5 (100)	
Socio economic status (*N* = 34)			
Above poverty line	2 (33.3)	4 (66.7)	*P* = 0.5,
Below poverty line	6 (21.4)	22 (78.6)	
Site of disease (*N* = 43)			
Pulmonary	9 (82)	32 (100)	*P* = 0.01
Extra pulmonary	2 (18)	0	
Regimen type (*N* = 33)			
New	5 (20.8)	19 (79.2)	*P* = 0.93
Previously treated	2 (22.2)	7 (77.8)	

*TB* Tuberculosis, *RNTCP* Revised National Tuberculosis Control Programme

### Qualitative findings

Seven key informant interviews and five patient interviews were conducted to explore the process of documentation of tobacco and alcohol use in the new TB treatment cards and to explore enablers and challenges in linking patients to tobacco cessation and alcohol abuse treatment services. Our document review included the TB treatment guidelines and the TB treatment cards. The details regarding addiction related information in the TB treatment card included if the patient is a current tobacco user or not, if yes type of tobacco use, linked to cessation or not and status of tobacco use at end of treatment which was assessed by the option Quit or Not Quit. Regarding alcohol abuse, the information includes if there is history of alcohol intake or not, if yes linked to deaddiction or not.

### Documentation of substance use

Health care providers overall felt that the inclusion of substance use related information in the new TB card was relevant, simple and beneficial for understanding the magnitude of the problem at the community and individual patient levels.

**“***It’s required both to control tuberculosis as well as the addiction problem. So under one umbrella we will be covering two things. It is simple and easier for the health worker as well as the participant also”. (*43 year old male medical officer)

On the other hand they revealed that there are multiple people involved in collecting information in certain places and designated staff in other places. This lack of standardization may affect the completeness and quality of the data collected.

*“ANM (Auxiliary Nurse Midwife) and I take mainly, because these direct questions are asked by the medical officers. My lab technician also takes”* (39 years old, male, medical officer)

### Process of implementing tobacco cessation services

Tobacco cessation services offered by health care providers include assessment of the level of addiction followed by counselling (general and TB specific), and, when indicated, referral to the psychiatric Department of the District Hospital.

Some medical officers mentioned that initially they provide counselling to the patients themselves and if there is no improvement, then they refer to specialists. *“For tobacco, No, we just advice and treat the case” (*43 year old male medical officer*). “We also counsel them as in what happens when you take tobacco and what are the ill effects of tobacco*”(37 years old, male, counsellor, Private centre).

Some practitioners gave examples of the content of their counseling sessions. *“Your lungs are already damaged and tuberculosis is going to eat up your lungs so much and if you further smoke then it will be totally destroyed and living a normal life will become difficult”. (*39 years old, male, Medical Officer).

Referral is either to public or private centers: *“Right now in District Hospital Psychiatry Ward they are doing [smoking cessation]. So we refer there or if they are ok then we refer to private also”* (35 years old, female, medical officer).

Tobacco cessation treatment involves initial assessment followed by treatment in outpatient basis.

*“During interview, we will try to know if he/she has dependence on tobacco and accordingly we treat with either nicotine gums or tablet Bupropion and counselling. Nicotine gums are available here freely”. (*male psychiatrist, 48yrs)

### Alcohol abuse treatment services

The review of TB treatment guidelines revealed that there are no clear cut guidelines as to how a patient with TB and alcohol abuse needs to be treated. The process of alcohol abuse treatment services as described by the health care providers include initial assessment, counselling (general and TB specific) and referral when indicated, based on the judgment of the treating physician. The management of TB patients with alcohol abuse does not appear to be standardized and no clear protocols and guidelines are followed. As one 35 year old medical officer described her typical practice; *“For alcoholics I usually send them for liver function tests initially. As this addition of drugs [TB] alter their liver function. Then again we counsel them. Most of them quit as they can’t take those [TB] tablets along with alcohol as it causes severe gastritis.”* The lack of clear guidance for assessing alcohol abuse and for linking to treatment services was also confirmed during our document review.

The health care providers were of the opinion that alcohol abuse treatment is a stepwise, graded approach and distinctively different from the smoking cessation procedure.

*“Alcohol is not like cigarette smoking. Cigarette smoking, if you stop one day you need to maintain it. But alcohol is not like that, if he takes today 1 quarter, in another 3 days he has to take 90ml and in another 3 days or 1- 2 weeks he has to take half of 90 ml. So it’s a gradual process in treatment.”* (Male medical officer, 39yrs)

As is the case with tobacco cessation, TB patients are referred to either public or private alcohol treatment services (“deaddiction” services as they are called in this context) according to the patient’s preference. The process of treatment as mentioned in the district hospital included initial assessment of dependence, counselling and detoxification. At both sectors the treatment is offered on in-patient basis.

*“From the history and assessment we will know if he is dependent on alcohol. If he is dependent on alcohol then we advise based on if he’s prepared to stop alcohol or not. If he is prepared to stop alcohol then it’s easy or we need to counsel him again regarding the ill effects of alcohol and how it can affect on his existing TB and accordingly we admit in the ward and detoxify them and we start medications.”* (48 year old male psychiatrist, district hospital)

### Enablers in providing tobacco cessation and alcohol abuse treatment services

The health care providers identified three broad enablers that facilitated linkage to tobacco cessation and alcohol abuse treatment services among patients. The themes were common for both conditions and included firstly family support, secondly will power and motivation from the side of the patients, and thirdly a perceived fear of complications.

The interviewees perceived that the role of family members in the tobacco cessation and especially the alcohol abuse treatment process was essential and tried to have them involved and engaged.

*“The family get them here as they think that alcohol and tobacco is also a major problem and the addiction also has to be treated.”* (37 years old, male, counsellor, private)

*“My family members also started irritating me they did so much Kirikiri (local slang for irritation). Then I felt very bad I decided to stop”.* (40-year-old male patient)

Some emphasis was given to perceived patient’s characteristics and especially what was perceived as “will power”. As a 52 years old female medical officer mentioned: *“Firstly as I earlier said will power of the patients. Some of the patients have good will power and will stop but some patients are so addicted and do not have the will power to stop”.*

Patient interviews also indicated the role of “will power” as one of the enablers especially with respect to alcohol abuse treatment. As a 40-year-old male patient mentioned *“It was my will power (garva). I just resisted it with the help of my will power”.*

The interviewed health providers referred to “fear of complications” as an enabler both among patients who use tobacco and the ones with alcohol abuse, however, it was not clear whether this is what they perceived as patient’s “own fears” or whether they felt they had to “enforce or instill” such fear as part of their intervention. The following quotes highlight this

*“One is the fear factor that because of these addictions they have got TB. So if we say that “Your tuberculosis will get cured only if you stop taking these things” then they might accept.”* (45 years old, male, medical officer)

*“They told me that “if you continue smoking beedi your disease will not get cured”. They told me that, “only if you stop smoking beedi it will get cured”. So I completely stopped beedi”* (60 year old male patient)

### Challenges in implementing tobacco cessation and alcohol abuse treatment services

**Fig. 3:**
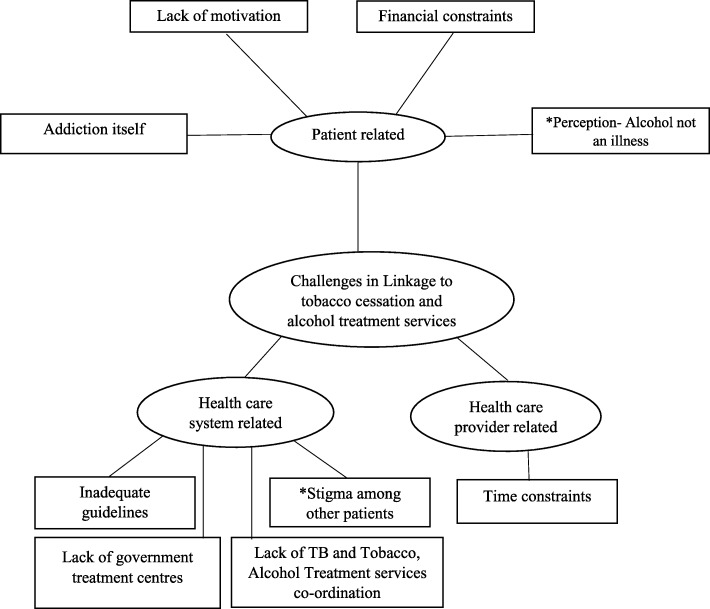
Perceived challenges in linkage of patients with TB to tobacco cessation and alcohol abuse treatment services. Key informant interviews and interview of patients with TB was conducted to explore the challenges in linkage of patients to the tobacco and alcohol abuse treatment services. Perceived challenges included lack of coordination between the TB treatment programme and tobacco cessation as well as alcohol treatment services, lack of motivation among patients and financial constraints

The challenges in implementing tobacco and alcohol abuse treatment services as perceived by the healthcare providers and the patients could be classified as being related to the patient, the health care provider or to the health system. Figure 3 shows a non-hierarchical model of the organizing and basic themes that emerged from the data.

### Patient related challenges

There was one overarching theme about the linkage to tobacco cessation services and this was patient’s lack of motivation that was expressed as reluctance to seek cessation services and even refusal to quit tobacco. Interestingly this theme was described by the health care providers while we were not able to elicit such information from the patient interview data (likely due to our inability to interview patients who refused linkage or participating in the interview)

“*Yeah, some will be chronic patients. They would be like “I cannot quit whatever maybe the treatment”. Then we send them to deaddiction centres. Some are reluctant to even go to the deaddiction centres.” (*35 years old female, medical officer)

The emerging challenges in linking patients to alcohol abuse treatment services were found to be slightly different. While the perceived patient’s lack of motivation was a common theme, in the case of alcohol abuse it was partly attributed to patient’s perception of alcoholism not being an illness. “*Sometimes they are in contemplation stage whether to stop alcohol or not or they may feel this is totally a different thing, it’s not an illness so it’s difficult to convince them.”* (48 years old, male, psychiatrist).

Financial constrains emerged as a challenge for both, especially for alcohol abuse requiring hospital admissions *“For deaddiction you have to pay here, it’s not free. Hence for people who are of lower socioeconomic status it becomes difficult.”* (52 years old, female medical officer, private facility).

Lastly withdrawal symptoms and the addiction itself (“temptation”) was found an additional challenge for patients who used alcohol compared to tobacco users. *“Especially in alcohol there is rum fit syndrome, that is if there is high intake of alcohol, even though we try our best they get this withdrawal symptoms and they have to take little amount of alcohol.” (*39 year old male medical officer).

A 40 year old male patient mentioned *“Initial two days it was very difficult. My hands were trembling, I was feeling like I need a drink, my head was aching, and morning as soon as I got up too I felt like drinking.”*

### Health care provider related challenges

Busy OPDs in some primary health centres pose a challenge to medical officers in properly managing patients, delivering quality counseling and advice and linking them to the tobacco cessation or alcohol treatment services.

“*Yeah sometimes in heavy OPDs (Out- patient department) it is difficult to talk for long”.* (35 years old, female, medical officer).

Even though during our document review we were able to locate guidelines regarding linking TB patients who are tobacco users to cessation services we found that there was lack of information among some health care providers, especially the ones working in primary health centres. *“No, right now we do not have any guidelines from Government of India”* (39 years old, male, medical officer).

The health care providers also felt that they lack training in treating patients with alcohol abuse, especially handling withdrawal symptoms.

“*We have some medications but we do not have so much knowledge to tackle the withdrawal symptoms”* (43 years old, male, medical officer).

### Health care system related challenges

The overall lack of formal coordination mechanisms between the TB programme and tobacco cessation services posed a challenge in linking patients between the two. Moreover, the extremely limited number (particularly the public ones) and geographic location of the few existing cessation centers made the navigation of patients difficult and especially for patients who had to travel; “*If they are coming from a distant place, these nicotine gums are not available in Taluka or PHC level, so they may find it difficult to come for follow up”* (48 yr. old male psychiatrist).

The existing integrated, one-stop service between HIV (Human Immunodeficiency Virus) and TB programmes was emerged as a model that juxtaposed the fragmented services offered by the TB programme and smoking cessation clinics; *“For example all TB patients have to do HIV testing and they are directly linked ie TB and ART (Antiretroviral Therapy) and follow up is there in ART centre. But such facility is not available for deaddiction or cessation services in government setup”* 52 yr. old female medical officer.

The existing guidelines do not offer clear and detailed guidance on linkage of TB patient to alcohol treatment services.

*“They have not mentioned anything in the guidelines about the cessation of alcohol and deaddiction. Only co morbid conditions have to be addressed is mentioned.”* (43yr old male medical officer)

There were no established mechanisms for linkage and thus they could not follow up referred patients.

*“We refer the patients and thus we are not in direct contact with them.”* (52 year old female medical officer)

Stigma and discrimination among other admitted patients towards the patients with TB was another challenge.

*“It’s difficult because if other patients come to know about the patients with TB or even HIV they do not talk or mingle with them, and start telling that “ I will leave the centre as there are TB patients here””* (37 year old male counsellor, private facility.)

## Discussion

This is the first mixed methods study in India that assessed the recently introduced documentation of alcohol and tobacco use among patients with TB treated under RNTCP and explored the enablers and challenges in linking them to tobacco cessation and alcohol abuse treatment services.

We found that almost three out of four TB treatment cards had the status of tobacco and alcohol use documented. Despite this being encouraging for a newly introduced set of data variables to be collected, there is definitely need for improvement. From the document review, it was observed that the addiction related information to be entered in the card is inadequate especially regarding alcohol use, leaving health care providers confused on whom to consider as current smoker or alcohol user. The card does not have the provision to include about brief advice on cessation given which has been mentioned in the TB treatment guidelines [15]. It is not clear from the card regarding who is to be linked to cessation services. The process for improvement should start by providing clear guidance on how to collect such data, what the definitions of the data variables are, who should collect and analyze and who should act upon them. In the case of alcohol abuse, our document review and interview data show that there is a need to immediately provide clinical and referral guidelines [9].

The proportion of tobacco users among the study population was high and consistent with the national figures according to GATS 2 survey, although this assessment was only among TB patients which may be the reason for the higher proportions, however it was lower when compared to other studies [5, 20–22]. About half of those who were tobacco users had documentation of the linkage status and more than one fourth of the tobacco users were not linked to the tobacco cessation services as per the new TB treatment cards. Since there is no documentation of the brief advice being offered as prescribed by the TB treatment guidelines, one cannot comment on whether this proportion is high or adequate.

In the present study males, patients with pulmonary TB, from rural area, belonging to lower socioeconomic status and new patients had higher linkage rates to tobacco cessation and alcohol abuse treatment services. Linkage to tobacco cessation services was higher among patients with TB who are linked to alcohol treatment services as well and this was found to be statistically significant.

Our interview data have shown that the inclusion of the addiction related information in the treatment card was considered to be beneficial for comprehensive management of TB by the health care providers which was also indicated in previous studies [22, 23]. The process of linkage to tobacco cessation services showed disparities as most of them followed different approach from counselling to referral.

We have observed a lack of standardization in managing and referring TB patients to substance use services. We encountered some counseling techniques and messages (as reported by the health providers themselves) that may not follow international standards by virtue of failing to take into account the individual patient sensitivities such as messages that may instill fear or helplessness. Some of the health care providers reported that there were not enough government tobacco cessation or alcohol treatment centres to which they could refer and this posed additional challenges in linkage and added financial constraints to the patients and their families.

Lack of coordination between the TB treatment programme and tobacco cessation and alcohol treatment services was considered a major challenge in effective implementation of the linkage services. A feasibility study on tobacco cessation done in Brazil also indicated similar findings [23]. The successful paradigm and model of the TB/HIV service integration could be considered in the case of substance abuse particularly for setting with high burden of TB and high rates of tobacco and alcohol use.

The solutions provided by the health care providers to improve the linkage system included establishment of government treatment centres for tobacco and alcohol treatment services preferably exclusive for TB patients, better TB treatment and tobacco, alcohol treatment services co- ordination and integrated services. The importance of alcoholism and TB drug’s adverse interactions was also stressed upon by the health care providers indicating the importance of alcohol treatment among TB patients. A study done in Poland also reports the association of alcoholism and adverse drug interactions among TB patients [7]. The health care providers also felt the need of training in implementing these services which was also stated in other studies [23, 24].

The present study gave an overview of the programmatic factors and challenges encountered in linking the TB patients to cessation services indicating the need for emphasis on monitoring, evaluation and better co-ordination between TB programme and tobacco and alcohol abuse treatment services. The study implies that there is a need for clear operational guidelines, accountability for the data entered, monitoring action on the linkage of patients with TB and substance use to treatment services, documentation of follow up status and outcome along with inclusion in TB reporting format.

### Limitations

All of the new TB treatment cards were not available for data collection as implementation of new TB cards was delayed till the month of April 2017 in few TUs of Dakshina Kannada district. This would have lead to an over or underestimation of the results. The lack of documentation posed a limitation to assess the association of various socio-demographic factors with the linkage services. Data could be validated for only a small subset of the total study population due to challenges in the two step obtaining informed consent.

## Conclusions

In conclusion, our study has shown that the documentation of the tobacco and alcohol use status was good but not universally done; hence this needs to be improved. Clear operational guidelines on linkage and treatment guidelines for health care providers to appropriately manage the patients with comorbidities are lacking. Lack of coordination between the TB treatment programme and tobacco cessation as well as alcohol treatment services was considered a major challenge in effective implementation of the linkage services, implying the need of these services to be strengthened and the health care providers and patients navigating between the services supported. Adequate monitoring and evaluation of the performance of linkage services are recommended.

## Additional file

Additional file 1: Interview guide. The Additional file 1 contains the interview guide which was used to collect qualitative data by patient interviews and key informant interviews of health care providers. (DOCX 17 kb)

## Abbreviations

ANM: Auxiliary Nurse Midwife
ART: Antiretroviral Therapy
BPL: Below Poverty Line
DMC: Designated Microscopy Centre
GATS: Global Adult Tobacco Survey
HIMS: Health Management Information System
HIV: Human Immunodeficiency Virus
KII: Key Informant Interview
MO: Medical Officer
OPD: Out Patient Department
PHI: Peripheral Health Institution
PI: Principal Investigator
RNTCP: Revised National Tuberculosis Control Programme
SD: Standard Deviation
TB: Tuberculosis
TCC: Tobacco Cessation Clinic
TU: Tuberculosis Unit
