# Supplementary material for: Are they there yet? Linkage of patients with tuberculosis to services for tobacco cessation and alcohol abuse – a mixed methods study from Karnataka, India
Source: BMC Health Serv Res. 2019 Feb 1;19:90. doi: 10.1186/s12913-019-3913-8 (PMC6359801; doi:10.1186/s12913-019-3913-8)
Supplement: Supplementary file 1 — Interview guide. The Additional file 1 contains the interview guide which was used to collect qualitative data by patient interviews and key informant interviews of health care providers. (DOCX 17 kb) [file 12913_2019_3913_MOESM1_ESM.docx]

**ADDITIONAL FILE-1**

**Key Informant Interview- Interview Guide** (18,19)

Following a brief introduction on the purpose of the interview to the participant, the PI will take a written informed consent from him/her for the interview and for the audio recording of the interview as well.

**Questions:**

1. Addiction related information is included in the new TB treatment cards. What is your opinion about this addition of information? (Probe: simplicity/ complexity, relevance, adequacy)
2. How do you go about collecting this information? (Probe: process, time taken, information shared)
3. What do you think are the advantages of including this information in the TB treatment card?
4. If you find out that the TB patient is a smoker and/ or alcohol user what do you do? (Probe: documentation, role in referral, follow up, counseling availability, feasibility)
5. What is the process you follow in treatment if a patient is smoker? (Probe: counselling, referral)
6. What is the process you follow in treatment if the patient gives history of alcohol use? (Probe: counselling, referral)
7. What are the aspects you consider helps you in providing or linking them to cessation / deaddiction services? (Probe: Patient’s receptiveness due to TB, effectiveness of physician provided counselling/ referral, DOTS centres and clinics tobacco free)
8. What are the challenges you face in providing or linking these patients to cessation/ deaddiction services? (Probe: training, limited time with patients, access to care, adherence, withdrawal symptoms, relapse after treatment completion)
9. According to your opinion how should tobacco and alcohol use in tuberculosis patients be tackled?
10. Additional remarks if any

the end of the interview PI will give a summary of the field notes taken and confirm the same from the participant. PI will complete the interview by acknowledging the time spared by the participant from his/her busy schedule.

**TB patient – Interview Guide**

Following a brief introduction on the purpose of the interview to the participant, the PI will take a written informed consent from him/her for the interview and for the audio recording of the interview as well.

**Questions:**

1. What is your experience with the process of TB treatment?
2. In the beginning of TB treatment where you asked about tobacco and/ or alcohol use?
3. If a tobacco user: what information regarding your tobacco use was asked at the time of diagnosis, during and end of treatment of TB? (Probe: duration, number of cigarettes/day, type of tobacco)
4. What advice regarding tobacco use was provided to you after diagnosis of TB? (general, TB specific)
5. Where you provided any cessation services for your tobacco use practices and if yes what were those services? (Probe: Cessation advice, referral to cessation centres)
6. If yes what was the process involved in receiving in these cessation services? (Probe: referral, waiting time)
7. How was your experience in receiving these cessation services? (Probe: access to cessation centres, waiting time, follow up)
8. Where these services helpful?
9. If yes how? (Probe: relief in symptoms)
10. If no why? and what were the problems you faced during this process? (access to care, withdrawal symptoms, drug interactions)
11. If history of alcohol intake present: what information regarding your alcohol use was asked at the time of diagnosis, during and end of treatment of TB? (Probe: duration, amount of alcohol, type of alcohol, eye opener drinking)
12. What advice regarding alcohol use was provided to you after diagnosis of TB? (Probe: general, TB specific)
13. Where you provided any deaddiction services for your alcohol use practices and if yes what were those services? (Probe: counselling, referral)
14. If yes what was the process involved in receiving in these services?
15. How was your experience in receiving these deaddiction services? (access, waiting period, health care provider’s approach)
16. Where these services helpful?
17. If yes how? (Probe: relief in symptoms)
18. If no why? and what were the problems you faced during this process? (Probe: access to care, waiting period, withdrawal symptoms, drug interactions)

At the end of the interview PI will give a summary of the field notes taken and confirm the same from the participant. PI will complete the interview by acknowledging the time spared by the participant from his/her busy schedule.
